# Perceived Cardiovascular Disease Risk Following Preeclampsia: A Cross-Sectional Study

**DOI:** 10.3390/healthcare11162356

**Published:** 2023-08-21

**Authors:** Nahed Ahmed Hussien, Nazia Shuaib, Zeinab Ali Baraia, Adel Omar Laradhi, Wenna Wang, Zhenxiang Zhang

**Affiliations:** 1 School of Nursing and Health, Zhengzhou University, Zhengzhou 450001, China; nahedahmed@nursing.suez.edu.eg (N.A.H.); naziashuaib@yahoo.com (N.S.); adlardhi2010@gmail.com (A.O.L.); wwn15290817551@163.com (W.W.); 2 Faculty of Nursing, Suez Canal University, Ismailia 41522, Egypt; zeinab_baraia@nursing.suez.edu.eg

**Keywords:** cardiovascular disease, risk, perception, preeclampsia, knowledge, awareness

## Abstract

Preeclampsia has been linked to an increased risk of cardiovascular disease (CVD), and the preeclamptic women were unaware of this link. Therefore, this study aims to assess women’s knowledge and perception of future CVD after preeclampsia. This study used a cross-sectional descriptive design. Two hundred and forty-six women with a preeclampsia history were recruited from the Al Salam MCH Center and Suez Canal University Hospital. Data were collected during March 2022 using a socio-demographic questionnaire, an Adapted Coronary Heart disease knowledge tool for preeclamptic women, and The Perception of Risk of Heart Disease Scale (PRHDS). Most women (96%) were unaware of the relationship between CVD and preeclampsia. The women had a low CVD knowledge level (10.26 ± 6.08) as well as a low perception of cardiovascular disease risk (37.15 ± 7.22). There was a significant positive correlation between CVD knowledge and CVD risk perception (r = 0.434, *p* = 0.000). This study found that preeclampsia survivors underestimated their CVD risk. Based on these findings, preeclamptic women should receive health education sessions on CVD risk and prevention from their nurses and obstetricians. The hospital pre-discharge plan must contain these sessions in written and electronic formats to help women remember and follow CVD risk reduction measures.

## 1. Introduction

Cardiovascular disease (CVD) remains the world’s leading cause of death and a significant source of disability, particularly stroke and ischemic heart disease. In 2020, CVD was responsible for the deaths of 19.1 million people worldwide [1]. The CVD burden globally has been steadily rising for decades, especially in low-income and developing nations. In Egypt, the mortality rate caused by cardiovascular disease accounted for 46.6% of overall deaths. Among these deaths, 32.4% of mortality was attributed to coronary artery disease (the most common and dangerous type of CVD), with a prevalence in women of 21.7% [2,3].

Women die from CVD up to four times more than breast cancer [4,5]. Women have specific female risk factors that increase their likelihood of developing CVD, such as a history of gestational diabetes, polycystic ovary syndrome, intrauterine growth restriction, preterm birth, miscarriage, use of hormonal contraception, menopause, and preeclampsia [6,7,8]. Preeclampsia (PE) is one of the hypertensive disorders during pregnancy (HDP) that usually starts after 20 weeks of pregnancy. It is characterized by high blood pressure ≥140/90 mmHg, edema, proteinuria, organ dysfunction, and uteroplacental dysfunction. Globally, PE affects 2–8% of all pregnancies, and more than 70,000 women lose their lives yearly because of preeclampsia [9,10]. Among Egyptian women, about 6–8% of all pregnancies are complicated by preeclampsia, and this number raises to as high as 15% in tertiary care facilities. Also, preeclampsia is still the primary cause of maternal mortality and represents 27.7% of the avoidable causes of maternal mortality [11,12].

Preeclampsia and CVD have the same pathological changes and risk factors, such as being overweight and having metabolic problems, high cholesterol, and insulin resistance. Preeclampsia is thought to occur because the placenta does not receive enough oxygen and blood flow due to a remodeling problem with spiral arteries. Also, the following oxidative stress causes an extreme systemic inflammatory reaction, which generates endothelial dysfunction and vasoconstriction, leading to high blood pressure throughout the body and poor blood flow to the organs. On the other hand, these pathological changes might not go away entirely after giving birth, increasing the chance of future heart problems later in life [13,14].

There is strong evidence that a history of preeclampsia is a significant risk factor for prospective cardiovascular and cerebrovascular disorders [4,15,16,17]. In 2019, a large cohort of 1.3 million women in the United Kingdom was studied to find out the relationship between hypertensive disorders of pregnancy, including preeclampsia, and the subsequent diagnosis of 12 different CVD. This study discovered that women who had ≥1 pregnancy complicated by preeclampsia had a risk ratio of 1.9 for any stroke (95% confidence interval 1.53–2.35), 1.82 (1.34–2.46) for peripheral events, 1.67 (1.54–1.81) for cardiac atherosclerotic events, 1.73 (1.38–2.16) for atrial fibrillation, 2.13 (1.64–2.76) for heart failure, 4.47 (4.32–4.62) for chronic hypertension, and 2.12 (1.49–2.99) for cardiovascular deaths [18].

Physicians still under-recognized preeclampsia when assessing CVD risk factors in women, even though the American Heart Association has included preeclampsia history as a risk factor for cardiovascular disease and suggested following a healthy lifestyle. A group of researchers studied how often internists ask about preeclampsia versus hypertension, smoking, and diabetes when evaluating cardiovascular disease risk factors at well-woman visits. Women were only asked about their preeclampsia history (23.6%) but were asked about smoking and diabetes (98.9%) and chronic hypertension (100.0%) [19].

Despite many studies linking preeclampsia and CVD risk, preeclamptic women have little or no awareness of their specific threat. Women with preeclampsia have expressed a strong desire for a more extended follow-up period that pays special attention to the physical and psychological effects of the condition [20,21,22]. Many preeclamptic women reported that neither their nurses nor obstetricians educated them on their risk of CVD, regular follow-up, or the benefits of adopting a healthy lifestyle [23]. Moreover, in 2019, a scoping review was conducted to determine how much women knew about the risk of CVD after HDP. Hypertensive disorders in pregnancy refer to conditions characterized by high blood pressure either before (chronic hypertension) or during pregnancy (pregnancy-associated hypertension). HDP is classified into four categories; gestational hypertension, preeclampsia/eclampsia, chronic hypertension, and chronic hypertension with superimposed preeclampsia [24]. Six of the seven studies found that women knew little or nothing about the link between CVD and HDP. Additionally, most of the women who were aware of the association did not obtain their information from their healthcare providers but from their own sources. In five of the six studies, healthcare providers were mainly unaware of the HDP-CVD link [21].

Appropriate risk perception is critical for either healthcare providers or preeclamptic women because it influences their health-related behavior. More follow-up visits after giving birth were recommended than the usual ones at six weeks. These visits should focus on finding and reducing future CVD risks [17]. If women with preeclampsia reasonably and scientifically understand their risk, they will follow the recommended risk-reduction procedures, minimizing their CVD risk [25,26]. Future research should focus on figuring out how much women and healthcare providers know about the risk of CVD that comes after complicated pregnancy with preeclampsia [27]. Therefore, this study aimed to assess women’s knowledge and perception of future cardiovascular disease risk after preeclampsia.

## 2. Materials and Methods

### 2.1. Study Design

This study utilized a descriptive cross-sectional design.

### 2.2. Study Setting

The study was conducted at Suez Canal University Hospital and AL–Salam MCH Center (outpatient obstetric and gynecological and family planning clinics), Ismailia, Egypt.

### 2.3. Participants

The participants were chosen using a predetermined set of criteria. The inclusion criteria include: (1) women who had a history of preeclampsia, including eclampsia and/or hemolysis, elevated liver enzymes, and low platelet count (HELLP) syndrome during their most recent pregnancy (3 months–5 years post-delivery); (2) women who were ≥18 years of age; (3) women who could read and write and had internet access. The exclusion criteria were that women had fetal demise in the most recent preeclampsia, chronic hypertension, or any cardiovascular disease [28].

### 2.4. Sampling and Sample Size

A purposive sampling technique was utilized to select 246 women with a history of preeclampsia from the target population by the principal investigator (N.A.H., nursing researcher) according to the inclusion and exclusion criteria. 

#### Sample Size Formula [29]

The sample size was calculated at a 95% confidence level Z score of 1.96. The margin of error (£) was 5% or 0.05. And *p* is the prevalence of the outcome variable, which was (0.20). Lastly, (*n*) = the calculated sample size = of 246 women [30].

$$n={\left[\frac{Z\propto /2}{E}\right]}^{2}\times \;P\;\left(1-P\right)$$

### 2.5. Research Instruments

#### 2.5.1. Socio-Demographic Questionnaire

This questionnaire was developed by the researcher based on literature and includes socio-demographic data (age, educational level, income, residence, body mass index (BMI), and preeclampsia history).

#### 2.5.2. Adapted Coronary Heart Disease (CHD) Knowledge Tool for PE Women

This tool was adapted from the original version of the Coronary Heart Disease Knowledge tool for women after taking the author’s permission to use it [31]. The current questionnaire contains 26 true and false questions (two were removed from the original questionnaire, four were added, and two were merged to be one question) after the pilot study. Scoring, correct response (true) = 1, incorrect response (false, I do not know) = 0. The scores range from 0 to 26; a high score indicates a higher knowledge level [28,31]. The Adapted CHD knowledge tool for PE women was translated into Arabic by back-translation [32]; Native Arabic speakers proficient in English performed the translation. First, they translated the questionnaire into Arabic. After that, the questionnaire was back-translated into English to ensure accuracy. Then, 10% of the study sample (who were excluded from the study) was used to test the instrument’s Arabic version, and the feedback was considered. Five experts evaluated the face and content validity of the adapted CHD knowledge tool for the PE women questionnaire. The questionnaire had a good face and content validity. Overall, Cronbach’s Alpha was 0.90.

#### 2.5.3. The Perception of Risk of Heart Disease Scale (PRHDS)

This scale was used to assess the CVD risk perception after obtaining the author’s formal permission to use the scale’s English and Arabic versions [33]. Through the domain identification process, risk perception was conceptualized into three dimensions: “dread risk”, “risk”, and “an unknown risk”. These dimensions of risk perception are intended to position persons on a scale ranging from low to high-risk perception. Each scale item has a four-point Likert scale response option ranging from one (strongly disagree) to four (strongly agree). The scale includes three subscales; dread risk, risk, and unknown risk. For the total score, item scores for each subscale are added together, and higher PRHDS subscale scores imply a higher risk perception of developing heart disease [33]. Across subscales, reverse scoring of these items (6,10–20) is required. The PRHDS had previously shown good construct validity, with an overall alpha scale of 0.80. The present study had a reliability coefficient of 0.86 on its scale.

Please see the Supplementary File S1 for details of the three research instruments.

### 2.6. Data Collection

The data were collected in March 2022; the researcher visited the study settings six days a week from 9 a.m. to 4 p.m. The women were selected based on inclusion and exclusion criteria and their willingness to participate. The researcher described the study’s goal to each woman individually. If the woman agreed to participate in the study, her written informed consent was taken. Each participant was required to answer questions about socio-demographics, cardiovascular disease knowledge, and risk perception of cardiovascular disease.

### 2.7. Data Analysis

The statistical package for social science (SPSS) version 23 was chosen to organize, revise, tabulate, and summarize the collected data. Based on the data type, the percentage, mean score degree, and standard deviation were used for the quantitative data, and the r-test (Correlation Coefficient) was utilized to test the correlation. The results were presented in appropriate tables.

### 2.8. Ethical Considerations

The Declaration of Helsinki guided the conduct of this study. This research was approved by the ethical committee of Zhengzhou University, China (IRB2021-134) and Suez Canal University, Egypt (144/2022). The authors Thanavaro et al. (2010) [31] and Ammouri & Neuberger (2008) [33] granted permission to use the study’s instruments. Data collection commenced after obtaining formal agreement from the Al Salam MCH center management teams and Suez Canal University Hospital. Written informed consent was obtained from participants. Participants can stop sharing their information and withdraw from the study at any point.

## 3. Results

### 3.1. Socio-Demographic Characteristics

There were 246 women in the overall sample. As shown in Table 1, the mean age of the studied women was 31.08 (SD ± 7.050) years; 99.2% were married, and 45.1% had secondary education. Among women, 46.7% were overweight, and 26% were obese. Regarding residence, 91.9% of women live in urban areas. Also, 37.4% of women reported insufficient outcomes.

### 3.2. Study Participants’ History of Preeclampsia

Table 2 shows that 99.2% of women had preeclampsia, and 30.1% of these women had it during their first pregnancy. Meanwhile, 96% of women were unaware that preeclampsia and CVD are linked. Only 2.4% of women learned from their obstetrician that preeclampsia makes them more likely to have CVD, while 1.6% learned about it from the Internet. Also, 95.2% of women said their healthcare providers did not teach them about CVD risk before being discharged from the hospital; see Table 2.

### 3.3. Study Participants’ Knowledge of Cardiovascular Disease

As presented in Table 3, the mean score of women’s knowledge of cardiovascular disease was low (10.26 ± 6.08). Most women (87%) were unaware that heart disease and stroke are the leading causes of death among women. When the women were asked about the general risk factors of cardiovascular disease, 68.7% of women correctly identified that advanced age increases the risk of coronary heart disease development. Also, 74, 70.3 and 69.5% of women correctly identified smoking, obesity, and high cholesterol levels as risk factors for heart disease. Furthermore, about half of women (48.4%) of women know that hypertension may cause heart disease.

Regarding specific CVD- female risk factors, 43.9% of women did not know they were at greater risk for heart disease after menopause than before, and only 8.1% of women knew that preeclampsia history increases the risk of stroke and coronary heart disease. Concerning signs and symptoms of heart attack, about two-thirds (69.9%) did not recognize pain in the neck, shoulder, arm, or back and dizziness as symptoms of a heart attack. At the same time, 53.3% of women did not identify shortness of breath, sweating, and nausea as heart attack symptoms; see Table 3.

### 3.4. Study Participants’ Perception of Cardiovascular Disease Risk

The results of the Perception of Risk of Heart Disease Scale are presented in Table 4, which shows that the mean score was relatively low (37.15 ± 7.22). The mean score for each of the three subscales was as follows: (12.97 ± 2.77) for the Dread Risk subscale, (11.46 ± 2.86) for the Risk subscale, and (10.77 ± 2.72) for the Unknown Risk subscale; see Table 4.

### 3.5. Correlation between Total Knowledge of Cardiovascular Disease and Total Perception of Cardiovascular Disease Risk

Table 5 reveals the correlation between CVD knowledge and perception of cardiovascular disease risk. There was a statistically significant positive correlation (r = 0.434, *p* = 0.000) between the overall cardiovascular disease knowledge and cardiovascular disease risk perception; see Table 5.

## 4. Discussion

This study assessed the women’s knowledge and perceived cardiovascular disease risk following preeclampsia. The results revealed that the studied women had low knowledge about cardiovascular disease and perceived themselves as at low risk of CVD. Furthermore, nearly all women (96%) were unaware of the link between CVD risk and preeclampsia. This is in accordance with the conclusion of two studies. They found that women with a preeclampsia history did not know about the link between preeclampsia and future CVD risk. However, these women were still interested in learning about it and motivated to live healthy lives [22,34]. Similarly, a scoping review was conducted in 2019 to measure women’s knowledge regarding CVD risk after preeclampsia, and six of seven studies reported that women had minimal or no knowledge about the relationship between CVD and hypertensive disorders during pregnancy. Most women who knew about the link had learned about it independently, not from their healthcare providers [20,21].

In contrast to the results of our study, other researchers found that nearly two-thirds of women who had preeclampsia and HDP knew that preeclampsia and CVD were linked. This discrepancy may be explained by the fact that in the previous studies, a more significant proportion of participants who were aware of the link between preeclampsia and CVD received information about CVD from the television, the Internet, or social media [23,35]. Meanwhile, in our study, only 1.6% of women reported learning about the link between preeclampsia and CVD from the Internet. In addition, it is worth noting that there is a lack of knowledge about CVD risk provided by obstetricians or nurses before hospital discharge (95.2% of women reported receiving no such education, and their obstetricians taught only 2.4 %); this could be illustrated by the shortage of obstetricians and nurses in comparison to the number of patients (women), resulting in an overwhelming workload. Consequently, their attention is mainly directed toward managing existing health issues and the ongoing management of preeclampsia following childbirth rather than providing health education about potential future health complications associated with a prior history of preeclampsia, such as CVD risk.

Regarding CVD knowledge level, the participants’ CVD knowledge was low (10.26 ± 6.08). Half of the studied women did not know the heart attack warning signs, such as chest pain, shortness of breath, sweating, dizziness, and pain in the neck, shoulders, arms, and back. This result could be attributed to their young age and education level, as half of the participants were under 30 and 45.1% of women had secondary education. Furthermore, they received no information on CVD before hospital discharge. This explanation is supported by another researcher who found that the level of education is significantly correlated with CVD knowledge [36]. Moreover, our finding is consistent with a Canadian survey to evaluate women’s perceptions, knowledge, and behaviors concerning their cardiovascular health. Less than half knew the most important warning signs of heart disease [37].

When women were asked about general risk factors for cardiovascular diseases, two-thirds of the studied women correctly identified that smoking, getting older, being overweight, and having high cholesterol were all risk factors for CVD. These results are in accordance with recent research studies; the first one found that women who had experienced preeclampsia had an in-depth understanding of modifiable risk factors for cardiovascular diseases, such as smoking and eating a diet high in cholesterol and saturated fat [28]. The second study conducted a self-administered online survey to evaluate knowledge and perception of CVD risk in young women during the reproductive years, which included women who had adverse pregnancy outcomes (HDP, gestational diabetes, preterm birth, and low birth weight infants) and women with normal pregnancies. They discovered that almost all participants from both groups (>96%) knew that obesity and smoking were CVD risk factors [35].

Contrary to our findings, fewer women identified smoking, high cholesterol, and hypertension as risk factors for heart disease [37]. This difference from our findings could be because the Egyptian government has been running awareness campaigns through media like TV to teach people about the dangers of smoking, being overweight, and having high cholesterol and their effect on heart health. In addition, over the past two years, the government has also been running early detection campaigns for chronic diseases like high blood pressure in maternal and child health centers.

On the other hand, most women in our study less correctly identified preeclampsia, low levels of female hormones, and menopause as risk factors for CVD. One possible explanation for this finding is that the preeclamptic women may believe their condition was temporary and would resolve itself after they gave birth to their babies. Also, most women in the study group were not yet in menopause, so they may not have paid much attention to the health problems after menopause [38]. Equally crucial is that all social media education in Egypt focuses solely on the primary risk factors for cardiovascular disease in both sexes.

Concerning cardiovascular disease risk perception, the study participants demonstrated a low-risk perception of CVD (37.15 ± 7.22). Recent studies found the same result; they reported a low level of women’s awareness about their high CVD risk after hypertensive disorders during pregnancy. Furthermore, others had a wrong perception of cardiovascular disease since half of the women with HDP and gestational diabetes thought their own risk of CVD was low. Women need to learn more about CVD risk and see it as a real health problem, not just a threat to their lives [21,28,34,35,37]. Contrary to what we found, preeclamptic women think they have a higher risk of CVD. This difference could be because almost two-thirds of the participants said they learned about CVD from their research, while 25.3% learned from their obstetrician, 13.2% learned from their general practitioner, and 6% learned from their midwives [39]. Meanwhile, in the current study, most of our participants did not receive pre-discharge health education from their healthcare providers. Moreover, only 2.4% of the 246 women learned about their risk from their obstetrician, and only 1.6% knew about it from the Internet. Our study and prior research [21,23] have identified a notable deficiency in health education provided by obstetricians and nurses to women with a history of preeclampsia regarding the potential cardiovascular disease risks. This insufficiency has contributed to women’s limited awareness of CVD risks, highlighting the importance of obstetricians’ and nurses’ roles in building preeclamptic women’s awareness of CVD risk. Hence, obstetricians and nurses must exhibit heightened attention in delivering health education to women, affording it the highest priority, formulating strategies for its implementation, and integrating it into the hospital discharge plan.

A further cause of the currently studied women’s poor perception of CVD risk is that women believe that CVD affects women less frequently than men, and participants were unaware of the specific female risk factors that put them at a higher risk than men. It is evident from the fact that only 13% of the currently studied women believed cardiovascular disease to be the leading cause of death among women; they did not view CVD as a significant threat to women. Also, 46% of American women cited cancer as the leading cause of illness in women and the greatest threat to their future health, while only 16% of these women cited CVD as their primary health concern [40].

Patients often do not have adequate knowledge about cardiovascular disease and exhibit a low perception of CVD risk [41]. This illustration supports the current study’s finding of a significant statistically positive correlation between CVD risk perception and cardiovascular disease knowledge; approximately half of the study participants lacked knowledge about signs and symptoms as well as female-specific risk factors, especially preeclampsia resulting in a low perception of CVD.

### Limitations

This study has some limitations; the current study was restricted to one city (Ismailia city) in Egypt; hence, results cannot be generalized equally to settings in other parts of the country. A large sample size will be needed for more validation of the findings. Furthermore, the utilization of a cross-sectional study design cannot determine the changes in women’s perception of CVD risk over time because the data were taken at a point in time.

## 5. Conclusions

This study concludes that women who have experienced preeclampsia do not know enough about the link between preeclampsia and the risk of CVD. There is a gap between the potential and real perceived risk after having preeclampsia; women think they are at low risk for cardiovascular disease later in life. Based on these results, midwives and obstetricians should discuss the risk of CVD with women with a history of preeclampsia. Before leaving the hospital, women with preeclampsia should attend at least one health education class about the causes, symptoms, and ways to avoid cardiovascular disease. Health education programs about CVD must be designed and available in printed and electronic forms to help women remember and adhere to cardiovascular disease risk reduction guidelines. Also, healthcare providers should encourage these women to regularly check their blood pressure, cholesterol, weight, and blood sugar.

## Figures and Tables

**Table 1:** Distribution of study sample according to their socio-demographic characteristics (*n* = 246).

Socio-Demographics	Variables	Frequency	Percent
Age Group Mean ± SD * 31.08 ± 7.050	≤20	15	6.1
	21:30	112	45.5
	31:40	86	35.0
	41:50	33	13.4
Education	Basic education	72	29.3
	Secondary education University education	111 63	45.1 25.6
Residence	Rural	20	8.1
	Urban	226	91.9
Marital Status	Married Divorced	244 2	99.2 0.8
Body Mass Index (BMI)	Normal: 18.5–24.9 Overweight:25–29.9 Obese: more than 30	67 115 64	27.3 46.7 26.0
Income	^#^ Sufficient	154	62.6
	Insufficient	92	37.4

* SD = Standard Deviation. ^#^ Sufficient = monthly income ≥ 4000 EGP.

**Table 2:** Distribution of study participants according to their preeclampsia history (*n* = 246).

History of Preeclampsia	Items	Number	Percent
Pregnancy complication	Preeclampsia	244	99.2
	Eclampsia	2	0.8
Preeclampsia occurrence in which pregnancy	First	74	30.1
	Second Third Fourth Fifth Sixth	72 46 30 16 8	29.3 18.7 12.2 6.5 3.2
Did the obstetrician discuss CVD risk with preeclamptic women before hospital discharge?	No	234	95.2
	Yes I do not remember	6 6	2.4 2.4
Do you know the relationship between CVD and Preeclampsia?	Yes No	10 236	4 96
If yes, what is the source of your knowledge?	Obstetric doctors	6	2.4
	Internet	4	1.6

**Table 3:** Distribution of study participants according to their knowledge about cardiovascular disease (*n* = 246).

CVD ^1^ Knowledge Items		Correct No. (%)	Incorrect No. (%)
1. Advanced age increases the risk of coronary heart disease development.		169 (68.7)	77 (31.3)
2. Heart disease related to heart artery blockages develops slowly over many years and can easily go undetected.		103 (41.9)	143 (58.1)
3. Women are more likely to have heart disease after menopause than before.		138 (56.1)	108 (43.9)
4. High cholesterol level may cause heart artery blockages.		171 (69.5)	75 (30.5)
5. Symptoms of heart pain or heart attack may include neck, shoulder, or arm pain, back pain, and dizziness.		74 (30.1)	172 (69.9)
6. African American women are more likely than white women to have heart disease.		21 (8.5)	225 (91.5)
7. High blood pressure may cause heart disease and stroke.		119 (48.4)	127 (51.6)
8. Some forms of heart disease may indeed result in stroke.		61 (24.8)	185 (75.2)
9. Symptoms of stroke are sudden numbness or weakness in the face, arm, or leg and sudden confusion.		64 (26.0)	182 (74.0)
10. Smoking may cause heart artery blockages.		182 (74.0)	64 (26.0)
11. Symptoms of heart pain or heart attack may include chest pain, chest tightness, and unusual fatigue.		127 (51.6)	119 (48.4)
12. Heart disease and stroke are the leading cause of women’s death.		32 (13.0)	214 (87.0)
13. A risk factor for heart disease that can be changed is heredity.		132 (53.7)	114 (46.3)
14. Reducing dietary red meat may prevent heart artery blockages.		65 (26.4)	181 (73.6)
15. Symptoms of a heart attack may include shortness of breath, sweating and nausea.		115 (46.7)	131 (53.3)
16. Reducing dietary cholesterol may prevent heart disease.		132 (53.7)	114 (46.3)
17. Stress may cause heart disease.		165 (67.1)	81 (32.9)
18. African American women are more likely than white women to die from a heart attack or stroke.		33 (13.4)	213 (86.6)
19. Obesity may increase the risk of heart disease.		173 (70.3)	73 (29.7)
20. There is no evidence that hormone therapy or replacement prevents heart disease.		44 (17.9)	202 (82.1)
21. A high-fat diet may cause heart artery blockages.		121 (49.2)	125 (50.8)
22. Low levels of some female hormones may increase heart artery blockages in women.		38 (15.4)	208 (84.6)
23. Routine exercise may prevent heart disease.		82 (33.3)	164 (66.7)
24. Diabetes increases the chance of having heart disease.		58 (23.6)	188 (76.4)
25. A history of preeclampsia increases the risk of coronary heart disease and stroke.		20 (8.1)	226 (91.9)
26. A family history of heart disease from a clogged heart artery may increase the risk of having heart disease.		86 (35.0)	160 (65.0)
Total CVD knowledge	Mean ± SD *	10.26 ± 6.08	

^1^ Cardiovascular disease, * SD = Standard Deviation.

**Table 4:** Distribution of study participants according to their perception of cardiovascular disease risk (*n* = 246).

Perception of CVD ^1^ Risk	Mean ± SD *
Dread Risk	12.97 ± 2.77
Risk	11.46 ± 2.86
Unknown Risk	10.77 ± 2.72
Total perception of CVD risk	37.15 ± 7.22

^1^ Cardiovascular disease, * SD = Standard Deviation.

**Table 5:** Correlation between total knowledge about cardiovascular disease and total perception of cardiovascular disease risk (*n* = 246).

**Total Knowledge** **of Cardiovascular Disease**	**Totalperception of** **Cardiovascular Disease Risk**	
	r	0.434 **
	Sig. (2-tailed)	0.000

** Correlation is significant at the 0.01 level (2-tailed).

## Data Availability

The datasets used and analyzed during the current study are available from the corresponding author upon reasonable request.

## Supplementary Materials

The following supporting information can be downloaded at: https://www.mdpi.com/article/10.3390/healthcare11162356/s1. In Supplementary File S1, you can find the study questionnaire, the Adapted Coronary Heart Disease (CHD) knowledge tool for PE women, and the Perception of Risk of Heart Disease Scale (PRHDS).
