# Supplementary material for: Perceived Cardiovascular Disease Risk Following Preeclampsia: A Cross-Sectional Study
Source: Healthcare (Basel). 2023 Aug 21;11(16):2356. doi: 10.3390/healthcare11162356 (PMC10454291; doi:10.3390/healthcare11162356)
Supplement: Supplementary file 1 [file healthcare-11-02356-s001.zip › healthcare-2472150-supplementary.pdf]

Supplementary file s1: Study questionnaires

1. **Socio-demographic data:**

Part 1: personal data:

1. **Age:** …………………
2. **Marital status:**

   1. Married ( ) 2. Divorced ( ) 3. Widow ( )

3. **Education:**

   1. Basic education ( ) 2. Secondary education ( )

   3. University education ( )

4. **Residence:**

   1. Rural ( ) 2.Urban ( )

5. **Monthly income:**

   1. Sufficient (monthly income ≥ 4000 L.E) ( ) 2. Insufficient ( )

6. **Height:** ………………
7. **Weight:** ……………..
8. **Body Mass Index (BMI):**……………..

- **Part 2 : History of preeclampsia :**

1. **Pregnancy complications:**

   1. Preeclampsia ( ) 2. Eclampsia ( )

2. **Preeclampsia occurrence in which pregnancy:**

   1. First ( ) 2. Second ( ) 3.Third ( )

   4. Fourth ( ) 5.Fifth ( ) 6.Sixth ( )

3. **Did the obstetrician discuss CVD risk with you before hospital discharge?**

   1. Yes ( ) 2. No ( ) 3. I do not remember ( )

4. **Do you know the relationship between CVD and Preeclampsia?**

   1. Yes ( ) 2. No ( )

5. **If yes, what is the source of your knowledge?**

1. Obstetric doctors ( )   2. Internet ( )

2. Knowledge of cardiovascular disease:

CVD[1] Knowledge Items	True	False	I do not know
1. Advanced age increases the risk of coronary heart disease development.			
2. Heart disease related to heart artery blockages develops slowly over many years and can easily go undetected			
3. Women are more likely to have heart disease after menopause than before.			
4. High cholesterol level may cause heart artery blockages.			
5. Symptoms of heart pain or heart attack may include neck, shoulder, or arm pain, back pain, and dizziness.			
6. African American women are more likely than white women to have heart disease.			
7. High blood pressure may cause heart disease and stroke.			
8. Some forms of heart disease may indeed result in stroke			
9. Symptoms of stroke are sudden numbness or weakness in the face, arm, or leg and sudden confusion.			
10. Smoking may cause heart artery blockages.			
11. Symptoms of heart pain or heart attack may include chest pain, chest tightness, and unusual fatigue.			
12. Heart disease and stroke are the leading cause of women's death.			
13. A risk factor for heart disease that can be changed is heredity.			
14. Reducing dietary red meat may prevent heart artery blockages.			
15. Symptoms of a heart attack may include shortness of breath, sweating and nausea.			
16. Reducing dietary cholesterol may prevent heart disease.			
17. Stress may cause heart disease.			
18. African American women are more likely than white women to die from a heart attack or stroke.			
19. Obesity may increase the risk of heart disease.			
20. There is no evidence that hormone therapy or replacement prevents heart disease.			
21. A high-fat diet may cause heart artery blockages.			
22. Low levels of some female hormones may increase heart artery blockages in women.			
23. Routine exercise may prevent heart disease.			
24. Diabetes increases the chance of having heart disease.			
25. A history of preeclampsia increases the risk of coronary heart disease and stroke. .			
26. A family history of heart disease from a clogged heart artery may increase the risk of having heart disease.			

3. Perception of Risk of Heart Disease Scale (PRHDS)

Item	Strongly disagree	Disagree	Agree	Strongly agree
1.There is a possibility that I have heart disease				
2. There is a good chance I will get heart disease during the next 10 years				
3. A person who gets heart disease has no chance of being cured				
4. I have a high chance of getting heart disease because of my past behaviors				
5. I feel sure that I will get heart disease				
6. Healthy lifestyle habits is something unattainable*				
7. It is likely that I will get heart disease				
8. I am at risk for getting heart disease				
9. It is possible that I will get heart disease				
10. I am not doing anything now that is unhealthy to my heart*				
11. I am too young to have a heart disease*				
12. People like me do not get heart disease*				
13. I am very healthy so my body can fight off heart disease*				
14. I am not worried that I might get heart disease*				
15. People my age are too young to get heart disease*				
16. People my age do not get heart disease*				
17. My lifestyle habits do not put me at risk for heart disease*				
18.No matter what I do, if I am going to get heart disease, I will get it*				
19. People who don't get heart disease, are just plain lucky*				
20. Heart disease has unknown cause*				

Note: * Reverse scoring of the item is required

Dread Risk (1,2,4,5,7,8,9), Risk (3,11,12,14,15,16), Unknown risk (6,10,13,17,18,19,20)
